# Social Support, Identity Affirmation, and Psychological Well-Being: A Developmental and Intersectional Comparison between Italian Cisgender and Non-Binary People with Bisexual Orientation

**DOI:** 10.3390/ijerph20043237

**Published:** 2023-02-12

**Authors:** Cristiano Scandurra, Concetta Esposito, Francesco Fantacci, Lorenzo Borrello, Vincenzo Bochicchio, Daniel Giunti, Paolo Antonelli

**Affiliations:** 1 Department of Neuroscience, Reproductive Sciences, and Dentistry, University of Naples Federico II, 80131 Naples, Italy; 2 Department of Humanistic Studies, University of Naples Federico II, 80133 Naples, Italy; 3 Centro Integrato di Sessuologia Clinica Il Ponte, 50136 Florence, Italy; 4 Department of Humanistic Studies, University of Calabria, 87036 Rende, Italy; 5 Department of Health Sciences, Psychology and Psychiatry Unit, University of Florence, 50121 Florence, Italy

**Keywords:** bisexual, minority stress, positive psychology, intersectionality, life course, social support, identity affirmation, well-being, health

## Abstract

Incorporating the perspectives of positive psychology, intersectionality, and life course into minority stress theory, this study aimed to examine the relationships between social support, identity affirmation, and psychological well-being among 483 Italian individuals with bisexual orientation, accounting for differences in gender identity (cisgender vs. non-binary) and age groups (young, early, and middle adult). A mediation model was tested in which identity affirmation served as a presumed mediator between social support and psychological well-being. We also examined whether gender identity and age group moderated the hypothesized associations. Multivariate ANOVA and multigroup mediation analyses were conducted. Results showed that (a) cisgender individuals had higher social support and psychological well-being than non-binary individuals, but not identity affirmation, which was higher in the latter group, (b) psychological well-being, but not social support and identity affirmation, differed between groups, with the youngest cohort reporting worse health than their elders, (c) identity affirmation mediated the relationship between social support and psychological well-being, (d) mediation was significant only in binary individuals (compared to cisgender), whereas no age differences were found. Overall, this study highlights the need to consider bisexual individuals as a nonhomogeneous population living multiple life experiences, especially when minority identities intersect.

## 1. Introduction

“Bisexual” is an umbrella term for people who engage in sexual or romantic relationships with more than one gender or are romantically, emotionally, and/or sexually attracted to more than one gender [1]. Bisexual people represent the largest group within the lesbian, gay, bisexual, transgender, queer, and other sexual and gender minority (LGBTQ+) population. For example, the LGBT+ Pride 2021 Global Survey [2] estimated that in a sample of 19,069 people aged 16–74 in 27 countries around the world, 4% identified as bisexual compared with 3% who identified as gay or lesbian. The frequency of people identifying as bisexual ranged from 9% in India to 1% in Turkey, Japan, and South Korea. In Italy, where the current study was conducted, the most recent demographic study by the National Institute of Statistics [3] indicated that of 7725 people aged 18–74, 2.4% identified themselves as gay and bisexual. However, this report did not disaggregate the data for these two populations.

Bisexual individuals are disproportionately affected by health disparities compared to heterosexual and lesbian and gay people [4,5]. The predominant theoretical framework in LGBTQ+ health research capable of explaining the psychosocial determinants of these health disparities is minority stress theory (MST), which posits that the stress experienced by people with a minority identity (i.e., due to poorer access to health, social, and economic resources than people who belong to a “majority” group, such as heterosexual and cisgender people) mediates the association between social minority status and health [6]. Although MST highlights the presence of significant resilience strategies that LGBTQ+ people use to protect themselves (e.g., social support, that is the function and quality of social relationships such as support received or perceived availability of help), recent research conducted within the framework of positive psychology [7,8] urges further emphasis on the resources and agency of this population by focusing on the positive aspects of LGBTQ+ identity to avoid propagating a portrayal of this population as powerless [9]. At the same time, recent research within the framework of intersectionality has increasingly emphasized the importance of viewing the bisexual population as nonhomogeneous [10,11]. Indeed, most research has focused on cisgender people with a bisexual orientation, but this is only one of the possibilities, as many transgender or non-binary people also have a bisexual orientation, and the lived experiences of these social groups can be very different [12,13,14]. Finally, one of the most recent scholarly trends is the incorporation of a life course perspective into LGBTQ+ health research [15,16,17], which examines life experiences in different generational cohorts who have diverse experiences depending on the social context in which they have lived or are living.

Thus, by integrating the frameworks of positive psychology, intersectionality, and life course into MST, the current study aimed to examine the relationships between some positive aspects of bisexual identity (i.e., perceived social support, identity affirmation, and psychological well-being) in a group of Italian bisexual individuals, taking into account both differences in gender identity (cisgender vs. non-binary) and developmental stages of the participants (i.e., young adults, early adults, and middle adults). In the following sections, we will first provide an overview of research on minority stress in the bisexual population. Then, we will present a possible integration of positive psychology, intersectionality, and life course frameworks into MST. Since our study focuses on Italy, we will present research findings on the Italian bisexual population at the end of the two sections.

### 1.1. Minority Stress and Health in Bisexual People

Although bisexual people represent a highly resilient community capable of successfully overcoming adverse life circumstances, they still face the social stigma associated with their minority status, which in turn impacts their health [4]. These health disparities can be explained using MST, a framework widely used to understand LGBTQ+ health determinants [6]. Namely, MST posits that social groups belonging to a sexual or gender minority identity experience social stigma due to their minority status. This type of stigma is a chronic, unique, and socially determined stressor that increases the risk of developing negative health outcomes. This occurs because social stigma causes various stressors (i.e., proximal stressors such as perceived discrimination, internalized stigma, concealment, and anticipated stigma) that act as mediators between stigma and health and increase the risk of developing negative health outcomes. Previous research has widely demonstrated that bisexual people are exposed to higher levels of minority stress which in turn puts them at greater risk for poor health (e.g., depression, obesity, etc.) than heterosexual people [1,18,19,20,21].

Compared to lesbian and gay individuals, bisexual people may experience unique stressors related to their bisexuality, commonly referred to as “biphobia” or “binegativity” [4]. Indeed, there are several stereotypes about bisexuality, often perceived as uncertainty about one’s sexual identity or as a form of promiscuity [22]. The bisexual community suffers from these stereotypes, widely spread among both heterosexual and lesbian and gay people, and this prevents them from fully utilizing their connection to the LGBTQ+ community as a protective factor against stigma [4,22]. However, this is not to say that bisexual people cannot activate resilience strategies to protect themselves from the negative health effects of antibisexual prejudice. In fact, previous research has shown that bisexual people are highly effective at promoting social adaptation by negotiating with their social environment, developing individual-level resilience strategies (e.g., identity affirmation), and increasing their access to community-level resources (e.g., bisexual community groups) [23,24,25].

Studies conducted with Italian bisexual people within the MST are consistent with the international literature and show that these people experience high levels of social stigma from both the heterosexual and lesbian and gay communities. Pistella et al. [26] found in a sample of 291 Italian lesbian, gay, and bisexual adolescents and young adults that bisexual subgroups had more difficulty coming out to their families than lesbian and gay subgroups. Verrastro et al. [27] found in a sample of 468 Italian lesbian, gay, and bisexual youth that bisexual subgroups were at higher risk for alcohol abuse than their lesbian and gay counterparts. Petrocchi et al. [28] found among 327 Italian lesbian, gay, and bisexual individuals that bisexual people exhibited lower levels of self-awareness, community connectedness, authenticity, and intimacy than their lesbian and gay counterparts. Monaco [29] found in a sample of 218 Italian bisexual individuals that they had great difficulty coming out because they felt they were not understood by others and argued that these individuals perceived the Italian context as strongly heteronormative. The most comprehensive study of minority stress among bisexual people in Italy was conducted by Scandurra et al. [30], who found that discrimination and internalized stigma were positively associated with poorer health, that internalized stigma mediated the association between discrimination and mental health problems, but that resilience did not moderate this latter association. Finally, Pistella et al. [31] found in a sample of 400 Italian bisexual and lesbian women that bisexual women had higher levels of internalized stigma, identity uncertainty, positive affect, identity self-awareness, and resilience compared with their lesbian counterparts.

As Rucco et al. [32] suggested, the high level of minority stress experienced by bisexual people in Italy may be mainly due to the fact that the social context and the legal system are not very supportive of this community. Indeed, the Italian legal system only recognized same-sex civil partnerships (but not equal marriage) in 2016, but it does not allow same-sex parents to adopt children. In addition, in 2020, Italian deputy Alessandro Zan proposed a bill to prohibit hate crimes based on gender identity and sexual orientation (i.e., the “Zan bill”), but it was rejected by the Italian Senate. Rucco et al. [32] assessed the impact of this structural form of stigma on the health and minority stress of a group of 299 Italian bisexual people by comparing two samples who completed online surveys before and after the rejection of the Zan bill and found that discrimination, anticipated and internalized binegativity, resilience, anxiety, and depression worsened in the second group and exacerbated the psychological well-being of Italian bisexual people.

### 1.2. Integrating Positive Psychology, Intersectionality, and Life Course Perspective into the Minority Stress Framework

Although MST is one of the most valuable frameworks for understanding LGBTQ+ health, three significant challenges should be considered. First, some scholars have recently criticized MST for its risk of generating negative narratives about LGBTQ+ people, although MST has played a critical role in depathologizing LGBTQ+ identity, strongly supporting the need for advocacy, and highlighting the presence of significant protective factors that can activate this population [8]. Nevertheless, most research in this area tends to describe LGBTQ+ people as stigmatized and powerless individuals who experience their sexual orientation and gender identity as catalysts of oppression. Second, most research addressing the bisexual community focuses on cisgender people (i.e., non-transgender, people whose gender identity matches their assigned sex at birth) and often does not consider that bisexual people can be simultaneously transgender, non-binary, genderqueer, or any other possible identity. Third, research has rarely considered that living as bisexual at different stages of life can have different effects on health as well as on how people experience their own identity and sexuality. Thus, one possible solution to these three challenges is to integrate three other theoretical frameworks into MST—rather than opposing MST [33]—namely, positive psychology [34], intersectionality [35,36], and the life course perspective [37].

#### 1.2.1. Positive Psychology

Positive psychology can be defined as a branch of psychology concerned with human flourishing (i.e., human strengths and virtues) by focusing on positive experiences (e.g., subjective well-being), positive traits (e.g., identity affirmation), and positive aspects of institutions and communities that can promote human development and psychological well-being [38]. In other words, it is a framework that goes beyond suffering and its alleviation [39]. When applied to the LGBTQ+ population, this framework considers the positive aspects of LGBTQ+ identities and emphasizes dimensions of strengths and resilience, including self-disclosure, coming out, identity affirmation, and connectedness to the LGBTQ+ community [25,40,41,42].

Studies conducted within a positive psychology framework and specifically targeting the bisexual population are scarce. However, the few studies that have been conducted seem to appreciate the use of this framework to understand the strengths of this population. For example, in a diary study with 91 bisexual adolescents, Flanders et al. [1] found that experiences perceived as positive were more related to the interpersonal level (e.g., perceived social support, belonging to a community, or normalization of bisexuality by peers) than to intrapersonal (e.g., internal attraction) or institutional (e.g., institutional support, institutional normalization of bisexuality, or activism) aspects. A study by Brownfield et al. [43] conducted with 36 bisexual individuals reported that coming out was perceived as a growth experience on both intrapersonal (e.g., living more authentically) and interpersonal levels (e.g., greater advocacy in daily interactions and improved relationships), as well as in terms of increased critical consciousness as it facilitates the development of an awareness of privilege and oppression.

In Italy, to our knowledge, the only study applying the positive psychology framework to bisexual people is that of Pistella et al. [31], who conducted their study on a group of lesbian and bisexual women (*n* = 400). The authors found that identity certainty and a positive identity are important protective factors against the negative effects of minority stress, as they can promote positive affect and social adjustment.

#### 1.2.2. Intersectionality

Intersectionality is a framework that dates back to third-wave feminism, coined by a Black scholar named Crenshaw [35] and currently widely used in psychological research [36]. Intersectionality helps critically analyze the oppressive experiences of people who have more than one stigmatized identity by considering gender identity, sexual orientation, ethnicity, religion, social class, and so forth. This perspective helps scholars view LGBTQ+ individuals as a nonhomogeneous social group and examine how multiple social identities can interact to represent different levels and axes of oppression or privilege. For example, cisgender identity, heterosexual orientation, white ethnicity, are all social identities associated with privilege and greater access to social and health resources.

As mentioned earlier, most of the research conducted with the bisexual community was focused on cisgender bisexual individuals and did not consider the possible intersectionality between gender identity and sexual orientation (we emphasize these two aspects because they were considered as main variables in the present work, but many other social identities should be considered within an intersectionality perspective, e.g., social class and ethnicity). In recent years, some studies have addressed this gap by analyzing possible differences in the experiences of cisgender and transgender people with a bisexual orientation. For example, in a sample of 488 cisgender and transgender bisexual people, Katz-Wise et al. [13] found that gender moderated the associations between bisexual-specific minority stress and physical health, with transgender people having worse health than cisgender women and men. Rahman et al. [14] found in a sample of 148 cisgender and transgender bisexual people that bisexual transgender participants had less access to health services (particularly trans women) and felt less comfortable with health care providers than cisgender bisexual people did. Dyar et al. [12] found in a sample of 360 bisexual individuals of diverse gender identities and ethnicities that bisexual minority-specific stress was associated with poorer physical health, internalizing symptoms, and substance use, but these associations were stronger among transgender individuals than cisgender individuals. At the same time, people of color showed stronger resilience than their White peers.

To our knowledge, no previous Italian studies have examined gender differences in bisexual people in terms of health, stress, or positive aspects of identity. Therefore, this study examined the associations between positive aspects (i.e., perceived social support and identity affirmation) and psychological well-being among cisgender and transgender adults with a bisexual orientation by testing for gender differences.

#### 1.2.3. Life Course

The life course perspective is a developmental theoretical framework that examines how differences in sociocultural contexts can affect people’s life experiences and identities as a function of their generational cohorts [37]. Because different generational cohorts share common social experiences in the form of social events and collective memories [44], the life course perspective makes it possible to assess the role of the individual- and group-level sociocultural contexts, such as stigmatization processes or cultural changes. In relation to the LGBTQ+ community, there have been many positive changes in recent decades. Some societies have become more supportive of this group of people [45,46], and some countries have granted new rights to LGBTQ+ individuals (e.g., marriage, same-sex civil partnerships, adoption, hate crimes laws, etc.).

Recent studies in LGBTQ+ health research have applied the life course perspective to both lesbian, gay, and bisexual people [15] and transgender individuals [16]. Both Meyer et al. [15] and Puckett et al. [16] found that sexual and gender identity milestones (e.g., self-identification as an LGBTQ+ person, coming out, etc.) occurred much earlier in younger cohorts than in older cohorts. However, neither minority stress nor health improved in the younger cohorts; on the contrary, minority stress remained unchanged, and mental health was better in the older cohorts. The latter datum was contrary to the authors’ expectations, who had expected significant improvements in both dimensions (i.e., minority stress and health) due to the supportive environment in which young LGBTQ+ people live. The authors concluded that improvements in the macrosocial context were unlikely to significantly impact stress and health processes because the cultural ideologies that lead to health disparities (i.e., homonegativity, heteronormativity, etc.) still represent systems of power that regulate social relationships and perpetuate differences in identity status.

To our knowledge, there are only two studies that have specifically used a life course perspective to assess the experiences of Italian LGBTQ+ people. Rosati et al. [47] found in a sample of 266 Italian LGBQ+ aged 20–80 that younger generations became self-aware, self-labeled, and came out earlier than older generations. Similarly, in a sample of 197 Italian transgender individuals, Scandurra et al. [17] found that younger participants identified as transgender and came out earlier than older cohorts. However, except for a few proximal minority stressors (i.e., negative expectations and disclosure), which were more prevalent among younger participants than older ones (as opposed to distal stressors, which did not differ between cohorts), younger participants had poorer mental health than their older counterparts. In essence, the study by Scandurra et al. [17] confirmed the findings of Meyer et al. [15] and Puckett et al. [16] by highlighting that changes in the social environment have a limited impact on both the stigma processes and mental health of Italian transgender individuals. Regardless, there are no studies that have examined age-related differences among Italian bisexual people in terms of their identity experiences and well-being.

### 1.3. The Current Study

Integrating positive psychology, intersectionality, and life course perspectives into MST, the current study aimed to explore the relationships between perceived social support, identity affirmation, and psychological well-being in a group of Italian individuals with bisexual orientation, considering potential differences in relation to two dimensions, namely gender identity (cisgender vs. non-binary) and age groups (young adults, early adults, and middle adults). The main variables analyzed are typically considered within the positive psychology framework as interpersonal (i.e., perceived social support), intrapersonal (i.e., identity affirmation), and health-related (i.e., psychological well-being) characteristics that capture positive aspects of LGBTQ+ identities. In addition, gender and age-related differences are typically considered in the context of intersectionality and life course perspectives, respectively.

Based on the work of Meyer et al. [15], Puckett et al. [16], and Scandurra et al. [17], we specifically hypothesized that (1) cisgender people would report higher levels of identity affirmation, perceived social support, and psychological well-being than non-binary individuals (Hypothesis 1), and that (2) younger generations would report higher levels of identity affirmation and perceived social support but lower levels of psychological well-being than older generations (Hypothesis 2). In addition, based on MST, which assumes that proximal identity dimensions act as mediators between interpersonal experiences (e.g., discrimination, rejection, etc.) and health [48,49,50,51], we also hypothesized that identity affirmation would mediate the association between perceived social support and psychological well-being (Hypothesis 3). In light of this last hypothesis, and to integrate the three theoretical perspectives used in the present work, we examined whether gender identity and age group influenced the hypothesized associations.

**Figure 1:**
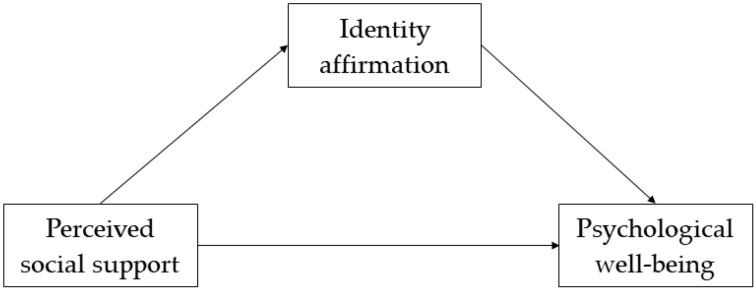
The hypothesized mediation model.

The hypothesized mediation model is shown in Figure 1.

## 2. Materials and Methods

### 2.1. Procedures

Data for the current study were collected through a web-based cross-sectional survey uploaded to Google. Participants were reached through online groups on bisexual and LGBTQ+ issues, primarily through Facebook. In addition, we asked potentially interested participants in the ads to share the survey with other potential participants, activating a snowball system for recruitment.

Participants were directed to the first page of the online survey by clicking on the link, where they received information about the researchers, objectives, study design, timing of completion, benefits, risks, and anonymity. Before starting the survey, participants had to give their consent by clicking on the button “I agree to participate.”

The project was approved by the ethical committee of the University of Naples (protocol number 15/2022, date of approval: 13 May 2022) and designed in respect of the principles of the Declaration of Helsinki on Ethical Principles for Medical Research Involving Human Subjects.

### 2.2. Participants

The survey was launched online between October and November 2022. Inclusion criteria were: (1) being at least 18 years old (the Italian age of consent), (2) self-identifying on the bisexual spectrum (bisexual, polysexual, pansexual, etc.), (3) living in Italy for at least 10 years, and (4) speaking the Italian language. A total of 497 participants completed the survey.

Age groups were based on the Schuler et al. [52] study, i.e., 18–25, 26–34, and 35–49 years, which are young adults, early adults, and middle adults, respectively. We could not include participants with 50+ years in the sample because only 4 met this criterion. In addition, 4 participants reported having an age < 18 years, and only 6 participants had a binary transgender identity (i.e., women, men, transman, or transwoman). Therefore, we removed 14 participants from the dataset, resulting in 483 individuals in the final sample.

Individuals in the final sample had a mean age of 27.14 years (standard deviation [SD] = 5.65, range = 18–49). Among them, 354 (55 males assigned at birth and 299 females assigned at birth) were cisgender, whereas 129 (26 males assigned at birth and 103 females assigned at birth) were non-binary. In addition, 282 (58.4%) had an education level ≥ college, and 476 (98.6%) were Caucasian. Sample characteristics are reported in Table 1. No differences between cisgender and non-binary individuals are detected on all sociodemographic characteristics (all *χ*^2^ > 0.05).

### 2.3. Measures

#### 2.3.1. Sociodemographic Characteristics

We collected the following sociodemographic variables: age, sex assigned at birth, gender identity (female, male, transgender females or males assigned at birth, bigender, genderfluid, nonbinary, genderqueer, pangender, gender questioning, agender, and other with specification), ethnicity, educational level (1 = ≤high school, 2 = ≥college), and actual stable partners (1 = no partner, 2 = one or more partners, with specification required). With respect to sexual orientation, we asked participants to specify their self-identification. Most of the participants self-identified as bisexual (71.2%) while others as pansexual (17.8%), queer (6.2%), demisexual (1.4%), asexual (1.2%), polysexual (0.4%), omnisexual (0.4%), or other (1.2%, e.g., greysexual, bi-curious, etc.).

#### 2.3.2. Perceived Social Support

Perceived social support was measured using the Multidimensional Scale of Perceived Social Support [53], a 12-item scale that assesses the extent of perceived support from different sources (family, friends, and significant others) on a 7-Likert scale (from 1 “very strongly disagree” to 7 “very strongly agree”). An example item is “There is a special person who is around when I am in need.” Higher scores reflect greater perceived social support. For parsimony, we used the total score, which *α* coefficient was 0.89.

#### 2.3.3. Identity Affirmation

Identity affirmation was assessed using the subscale “identity affirmation” of the Bisexual Identity Inventory [24]. This 6-item subscale measures the extent of comfort and pride with one’s bisexual identity on a 7-item Likert scale (from 1, “strongly disagree” to 7, “strongly agree”). An example item is “I am proud to be bisexual.” Higher scores reflect greater identity affirmation. The *α* coefficient for the current sample was 0.86.

#### 2.3.4. Psychological Well-Being

Psychological well-being was measured using the Psychological Well-Being Scale [54], an 84-item scale measuring different dimensions of psychological well-being that correspond to 6 subscales, i.e., autonomy, environmental mastery, personal growth, positive relations, purpose in life, and self-acceptance. The response options ranged from 1 (“strongly disagree”) to 6 (“strongly agree”). Example items are “I am not afraid to voice my opinions, even when they are in opposition to those of most other people” or “In general, I feel I am in charge of the situation in which I live.” Higher scores reflect greater psychological well-being. For parsimony, we used the total score, which *α* coefficient was 0.96.

### 2.4. Statistical Analyses

First, we conducted analyses to provide descriptive information about the sample and partial correlations between variables (i.e., perceived social support, identity affirmation, and psychological well-being) controlled for age and split by gender identity (cisgender vs. non-binary). Furthermore, the univariate normality was assessed by examining skewness and kurtosis values for each variable in the study, in the overall sample, and splitting by age and gender identity groups. No variables approached skewness > |3| or kurtosis > |10|, indicating that data followed a normal distribution [55].

Two analyses of variance (ANOVAs) were then conducted to assess gender (cisgender vs. nonbinary) and age (young adult vs. early adult vs. middle adult) differences in perceived social support, identity affirmation, and psychological well-being. In the ANOVAs, gender and age were included separately as fixed factors. The effect size was assessed using eta-squared (*η*^2^, small effect = 0.01, medium effect = 0.06, and large effect = 0.14). In the ANOVA regarding age groups, the Tukey’s post hoc test was used to assess between-group differences.

Finally, to test the hypothesized mediation model with gender identity (cisgender vs. non-binary) and age (young adults, early adults, and middle adults) groups as moderating variables, two multiple-group path analyses were performed. For each grouping model, two nested models were considered for testing whether differences in the structural parameters across groups were statistically significant: a baseline model, in which structural parameters were freely estimated across groups, and a fully constrained model, in which the paths were constrained to be equal across groups. When testing the equivalence across age groups, all the effects were controlled for gender identity groups and vice versa. For model comparison, the chi-square difference test (Δ*χ*^2^) was used, and modification indices were evaluated in order to improve the model fit. The effect size for the indirect effects was estimated using a bias-corrected bootstrapping approach (*n* = 1000) to obtain 99% confidence intervals (CIs). Other fit indices used to estimate the fit of the models were the comparative fit index (CFI), root mean square error of approximation (RMSEA), and standardized root mean square residual (SRMR). As cut-offs, we considered CFI ≥ 0.95, RMSEA ≤ 0.06, and SRMR ≤ 0.08 as indicators of the model’s acceptable fit to the data [56].

A *p*-value probability level of <0.05 was adopted for all statistical tests.

## 3. Results

### 3.1. Descriptive Statistics and Partial Correlations

Means, SDs, ranges, and partial correlations (controlled for age) between perceived social support, identity affirmation, and psychological well-being are shown in Table 2.

The results indicated that in cisgender individuals with a bisexual orientation, all variables were positively correlated. On the contrary, in non-binary individuals with a bisexual orientation, both perceived social support and identity affirmation were positively associated with psychological well-being, while no significant correlation was found between perceived social support and identity affirmation.

### 3.2. Associations of Perceived Social Support, Identity Affirmation, and Psychological Well-Being with Gender Identity and Age Groups

Regarding Hypothesis 1, the ANOVA revealed significant differences between cisgender and non-binary people with a bisexual orientation on all the psychological dimensions considered (i.e., perceived social support, identity affirmation, and psychological well-being).

Specifically, as shown in Table 3 and as hypothesized, cisgender individuals reported higher mean scores for perceived social support and psychological well-being than non-binary individuals. Contrary to our hypothesis, non-binary individuals reported higher mean scores on identity affirmation than their cisgender counterparts. All differences were of small effect size.

Furthermore, as shown in Table 4, in contrast to Hypothesis 2, no differences were found between age groups in perceived social support and identity affirmation. Instead, as hypothesized, mean scores of psychological well-being differed significantly between age groups, with the youngest generation reporting poorer psychological well-being than the older, with a small effect size. Specifically, Tukey post hoc comparisons showed that the mean difference in psychological well-being differed significantly between young adults and early adults (MD [mean difference] = −2.43, SE [standard error] = 0.89, *p* = 0.021) and between young adults and middle adults (MD = −3.92, SE = 1.45, *p* = 0.021), but not between early adults and middle adults (*p* > 0.05).

### 3.3. Multiple-Group Mediation Models

#### 3.3.1. Gender Identity as Grouping Variable

**Figure 2:**
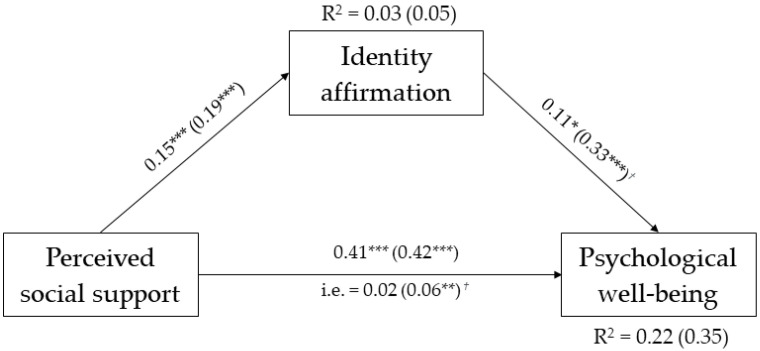
Multiple group mediation model for gender identity. Standardized path coefficients are reported. Parameters for non-binary trans are shown in brackets, † stands for significant differences between the groups. ** *p* < 0.01 *** *p* < 0.001. Cohort groups are not included in the figure for the sick of simplicity.

The comparison between the freely estimated and the fully constrained models in which all the structural parameters were held equivalent across the gender identity groups indicated that imposing the equality constraints led to a non-significant difference in the model fit, Δ*χ*^2^ (9) = 12.280, *p* = 0.20, CFI = 0.98, RMSEA = 0.04, 90% CI [0.00, 0.09], SRMS = 0.05. However, modification indices suggested that releasing the constrained path linking identity affirmation with personal well-being would improve the model fit. This modification resulted in a significant improvement in the model fit, *χ*^2^ (8) = 3.128, *p* = 0.93, CFI = 1.00, RMSEA = 0.00, 90% CI [0.00, 0.02], SRMS = 0.02; Δ*χ*^2^ (1) = 9.152, *p* < 0.01. All paths and standardized coefficients for the final model are presented in Figure 2.

As can be observed, perceived social support was positively associated with identity affirmation and personal well-being, and these associations were equal across groups; identity affirmation had a significant and positive effect on personal well-being in both gender identity groups, but this relationship was stronger in the non-binary group. The examination of the indirect effects highlighted that identity affirmation mediated the relationship between perceived social support and personal well-being only in the non-binary group, *β* = 0.06, *p* < 0.01, 99% CI [0.02, 0.13]. The percentage of variance explained for identity affirmation was 3% and 5% in the cisgender and non-binary groups, respectively, and the percentage of variance explained for personal well-being was 22% and 35% in the cisgender and non-binary groups, respectively.

As for the control variables, both early and middle adults showed higher levels of personal well-being compared to young adults (*β*s = 0.13, *p*s < 0.01). No other significant effect was found.

#### 3.3.2. Age Groups as Grouping Variable

**Figure 3:**
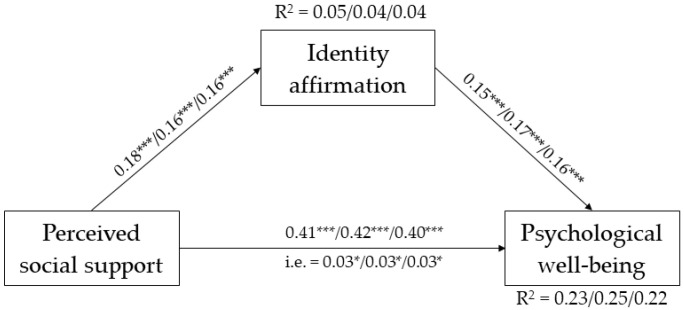
Multiple group mediation model for age cohorts. Standardized path coefficients are reported (young adults/early adults/middle adults). * *p* < 0.05 *** *p* < 0.001. Gender identity groups are not included in the figure for the sick of simplicity.

The fully constrained model in which all paths were held equivalent across age groups did not fit worse than the unconstrained model, Δ*χ*^2^ (12) = 5.904, *p* = 0.92, CFI = 1.00, RMSEA = 0.00, 90% CI [0.00, 0.03], SRMS = 0.06. Furthermore, no modification indices were suggested, supporting that the hypothesized effects were equal across groups. More specifically, we found that perceived social support was positively associated with identity affirmation and personal well-being, and identity affirmation had a significant and positive effect on personal well-being. The standardized coefficients are reported in Figure 3.

The examination of the indirect effects revealed a significant mediating effect of identity affirmation in the relationship between perceived social support and personal well-being, *β* = 0.03, *p* < 0.01, 99% CI [0.01, 0.06]. The percentage of variance explained was between 4% and 5% for identity affirmation, and between 22% and 25% for personal well-being.

As regards the effect of gender identity on the study’s variables, we found that non-binary individuals reported higher levels of identity affirmation, *β* = 0.18, *p* < 0.001, lower perceived social support, *β* = −0.14, *p* < 0.01, and lower personal well-being, *β* = −0.12, *p* < 0.01, compared to cisgender individuals.

## 4. Discussion

Informed by the incorporation of positive psychology, intersectionality, and life course perspectives into MST, this study was aimed at exploring the relationships of positive interpersonal (i.e., perceived social support) and intrapersonal (i.e., identity affirmation) factors with psychological well-being in a group of Italian bisexual people, accounting for differences in gender identity (cisgender vs. non-binary) and age groups (young, early, and middle adult). In general, our findings supported the main hypothesis concerning the relationships investigated—i.e., identity affirmation mediates the association between perceived social support and psychological well-being—and, at the same time, showed some significant differences both in terms of gender identity and age groups, confirming the scientific utility of integrating more perspectives in the LGBTQ+ health research.

Before commenting on the main findings, we should first mention that most transgender participants in the current study self-identified with a nonbinary identity, which does not allow us to assess possible differences between binary and nonbinary identities. The composition of our sample reflects the current trend in the transgender population, which increasingly identifies with a non-binary gender concept, particularly among the younger generation [57,58,59]. Indeed, our sample is not evenly distributed across age groups, with a higher proportion of participants in the younger cohorts (particularly the young and early adult groups). Thus, this finding simultaneously reflects a limitation of the study and a picture of contemporary society in which gender identifications are moving further away from the traditional system of gender division.

Regarding the differences between cisgender and non-binary people with bisexual orientation in all psychological dimensions considered (i.e., perceived social support, identity affirmation, and psychological well-being), the results partially confirmed our first hypothesis. Indeed, we found that cisgender individuals reported higher mean scores for perceived social support and psychological well-being than non-binary individuals, but not for identity affirmation, which was higher in the latter group than in cisgender peers. The first set of findings can be explained by the assumption that, within an intersectional paradigm, cisgender individuals have a more normative identity than non-binary individuals and therefore have more access to sources of social support than non-cisgender peers, which is strongly associated with psychological well-being and health [12,13,14]. Similarly, the second finding (i.e., higher levels of identity affirmation among non-binary individuals than among cisgender counterparts) can be explained by the application of the intersectional paradigm, but also by MST. Namely, identity affirmation involves the experience of being recognized by others who validate and affirm one’s identity rather than denying it [60], and these experiences contribute to developing a coherent sense of self as well as pride and comfort in one’s identity [24]. Identity affirmation is a critical factor, especially for people who experience a discrepancy between internal and external views of self, such as transgender and gender nonconforming people [60]. Because non-binary people with bisexual orientation generally have more visible and less normative identities and/or aspects than cisgender peers with bisexual orientation, it is likely that they also have more opportunities to disclose their identities to others and therefore negotiate their feelings and experiences with the social context to a greater extent than cisgender peers. While this can be stressful and might put these individuals at risk of denial and rejection [61], it can also increase the opportunity to reflect on and reinforce one’s identity, which positively impacts health [60,62]. However, what has been reported thus far relates to bisexual identity, as the scale we used was specific to bisexual identity affirmation. Future studies could examine the interplay between bisexual and transgender (or non-binary) identity affirmation and assess whether or not both have equal salience in the health processes of this population.

Regarding differences between age groups on the main variables, we found that only psychological well-being differed between groups, with younger cohorts reporting worse health than older cohorts. In contrast, there were no differences between groups in perceived social support or identity affirmation. These findings are consistent with previous studies reporting that, although identity milestones (such as coming out) tend to occur earlier in younger cohorts than in older cohorts, minority stressors generally did not differ between groups. In comparison, mental health was better in older cohorts of LGBTQ+ individuals than in younger ones [15,16,17]. It is widely recognized that age is a protective factor against negative mental health outcomes, both in general [63,64,65] and in LGBTQ+ populations [66,67]. Thus, following the MST and life course perspectives, it is reasonable to assume that older people had more time and social opportunities to learn adaptive coping strategies to deal with minority stressors and to strengthen their resilience strategies, which in turn support the development of health processes. However, we had expected to see differences between age groups in perceived social support and identity affirmation, but this was not the case. It is possible that the relatively small age range in our sample was not very sensitive to these differences and that we would have found significant differences if we could have included both adolescents and boomers (i.e., approximately people aged 55 years and older). Future studies could take this problem into account and recruit more diversified samples in terms of age, repeat our analyses, and retest possible differences between groups.

Finally, regarding the results of the mediation model, we found that identity affirmation acts as a significant mediator between perceived social support and psychological well-being. However, when the possible effect of gender and age was considered, we found that the mediating role of identity affirmation was independent of age but not gender, as mediation was significant only for non-binary individuals. This finding can be explained by both the positive psychology framework and MST, which postulates that the role of intrapersonal factors (e.g., identity affirmation) explains why interpersonal dimensions (e.g., perceived social support) influence mental health and well-being [49,50,51,68]. Briefly, being the minority stress socially-based, positive social experiences (such as the benefits of social support) would increase positive identity dimensions, which in turn promote human flourishing. As noted earlier, identity affirmation is a critical dimension for LGBTQ+ people [60], and our findings appear to show this is the case across all developmental stages of life. However, as reported in previous studies [60,69] and explained earlier, identity affirmation is particularly crucial for people with less normative identities, such as non-binary individuals. This does not mean that identity affirmation is irrelevant for cisgender individuals, but cisgender people would likely benefit more from other types of positive identity dimensions, such as authenticity, self-awareness, freedom from social labels, personal growth, etc. [25,70]. Future studies may therefore consider the need to differentiate intrapersonal identity factors that promote LGBTQ+ adjustment and well-being, particularly when structuring psychological interventions that promote health.

This study has several important limitations that should be considered when interpreting the results. First, it was a cross-sectional study using mediation analysis, which is more appropriate for longitudinal studies. Furthermore, among the theoretical frameworks used in the current study, the life course perspective would also require a longitudinal study design to assess the developmental trajectories of LGBTQ+ health and identity status in specific sociocultural contexts, whereas we were only able to capture a picture of participants at one point in their lives. Future research should therefore use longitudinal studies to identify cause-effect relationships among study variables and interpret results in terms of historical change. Second, some limitations are associated with the composition of the sample. For example, we could not assess the magnitude and relationships among the psychological dimensions studied among individuals aged 50 years and older and among adolescents (under 18 years of age). The same problem arises with ethnicity (i.e., almost all participants were Caucasian), binary transgender identity (i.e., almost all participants were non-binary), and community size (urban vs. rural). Future studies should strive to recruit a more diverse sample in terms of age, ethnicity, gender identity, and social contexts and assess potential differences based on these identity and social characteristics within an intersectional paradigm. Third, we have examined very few positive aspects of bisexual identity, while many other characteristics (e.g., authenticity, self-esteem, community connectedness, etc.) should be considered in future studies to paint a picture that is closer to the reality of this diverse population.

## 5. Conclusions

This is the first study to incorporate more than one theoretical framework commonly used in LGBTQ+ health research (i.e., positive psychology, intersectionality, and life course perspectives) into MST to understand the experiences of a group of Italian bisexual people. Overall, our results have shown that, especially for non-binary people with bisexual orientation, identity affirmation is a personal trait that can be reinforced by positive social relationships and that, in this way, acts as a driving factor for health and well-being. Thus, this study sheds light on the need to consider bisexual people as a nonhomogeneous population living diverse life experiences, especially in the presence of intersecting minority identities. Future studies could extend our findings by recruiting a more diversified sample in terms of age and ethnicity and by examining other psychosocial determinants of health and bisexual identity dimensions. In addition, it would be interesting to compare our findings with different sociocultural contexts, for example, individualistic and collective cultures conducting culturally sensitive research in LGBTQ+ health [71].

## Figures and Tables

**Table 1:** Demographics of the sample.

	Total Sample (*n* = 483)	Cisgender (*n* = 354)	Non-Binary (*n* = 129)	
	*n* (%)	*n* (%)	*n* (%)	*χ* ^2^
Age				
18–25 years	213 (44.1)	150 (42.4)	63 (48.8)	1.63
26–34 years	219 (45.3)	165 (46.6)	54 (41.9)	
35–49 years	51 (10.6)	39 (11)	12 (9.3)	
Sex assigned at birth				
Male	81 (16.8)	55 (15.5)	26 (20.2)	1.44
Female	402 (83.2)	299 (84.5)	103 (79.8)	
Ethnicity				
Caucasian	476 (98.6)	349 (98.6)	127 (98.4)	0.01
Non-Caucasian	7 (1.4)	5 (1.4)	2 (1.6)	
Education				
≤High school	201 (41.6)	143 (40.4)	58 (45)	0.81
≥College	282 (58.4)	211 (59.6)	71 (55)	
Stable partner				
No	209 (43.3)	152 (42.9)	57 (44.2)	0.06
One or more	274 (56.7)	202 (57.1)	72 (55.8)	

**Table 2:** Descriptive statistics and partial correlations between perceived social support, identity affirmation, and psychological well-being in cisgender and non-binary people with bisexual orientation.

	1	2	3	*M* ± *SD*	Ranges
1. Perceived social support	—	0.10	0.51 ***	5.27 ± 1.13	1–7
2. Identity affirmation	0.18 **	—	0.38 ***	6.06 ± 1.05	1–7
3. Psychological well-being	0.42 ***	0.19 ***	—	56.47 ± 9.39	14–84

Note: *M* = Mean, *SD* = Standard Deviation. Cisgender individuals’ scores are below the diagonal, and non-binary individuals’ scores are above the diagonal. *** *p* < 0.001, ** *p* < 0.01.

**Table 3:** Means comparisons of perceived social support, identity affirmation, and psychological well-being based on gender identity.

	Gender Identity					
	Cisgender (*n* = 354)		Non-Binary (*n* = 129)			
	*M*	*SD*	*M*	*SD*	*F*	*η^2^*
Perceived social support	5.36	1.09	5.02	1.17	8.79 **	0.02
Identity affirmation	5.98	1.09	6.27	0.87	7.20 **	0.01
Psychological well-being	57.34	9.14	54.09	9.68	11.54 **	0.02

*M* = Mean, *SD* = Standard deviation, *η*^2^ = eta-squared. ** *p* < 0.01.

**Table 4:** Associations of perceived social support, identity affirmation, and psychological well-being with age groups.

	Age Cohorts							
	Young Adults (18–25 Years) (*n* = 213)		Early Adults (26–34 Years) (*n* = 219)		Middle Adults (35–49 Years) (*n* = 51)			
	*M*	*SD*	*M*	*SD*	*M*	*SD*	*F*	*η^2^*
Perceived social support	5.21	1.14	5.33	1.10	5.26	1.19	0.55	<0.01
Identity affirmation	6.17	0.99	5.95	1.07	6.07	1.12	2.46	0.01
Psychological well-being	54.96 _a_	9.49	57.39 _b_	8.71	58.88 _b_	10.88	5.59 **	0.02

*M* = Mean, *SD* = Standard deviation, *η*^2^ = eta-squared. Means not sharing the same subscript are significantly different from one another. ** *p* < 0.01.

## Data Availability

Anonymized data will be made available upon reasonable request to the corresponding author.
